# Pharmaceutical innovation and access to financial markets

**DOI:** 10.1371/journal.pone.0278875

**Published:** 2022-12-20

**Authors:** Benjamin M. Blau, Todd G. Griffith, Ryan J. Whitby

**Affiliations:** Department of Economics and Finance, Jon M. Huntsman School of Business, Utah State University, Logan, Utah, United States of America; Universiti Malaysia Sabah, MALAYSIA

## Abstract

While prior research shows that pharmaceutical innovation generates measurable benefits for society, over the last 70 years, the innovative activities of pharmaceutical firms have dramatically declined. In this study, we develop and test the hypothesis that to innovate, pharmaceutical firms must have access to capital through well-developed financial markets. Using a broad cross-country sample from 1989 to 2016, we document that financial market development is associated with greater levels of pharmaceutical innovation. To draw stronger causal inferences, and to overcome potential endogeneity issues, we use both instrumental variable and difference-in-difference analysis. Our results suggest that access to capital markets plays a vital role in pharmaceutical innovation.

## 1. Introduction

Among the industries that experience high rates of innovation, perhaps none has more of an effect on society than the pharmaceutical industry. An important illustration is the industry’s response to the recent COVID-19 pandemic (see e.g., [1–3]). Prior research also shows that pharmaceutical innovation leads to lower healthcare costs, fewer hospital visits, and enhanced quality of life. In particular, [4] shows that for every 100 new drugs produced, aggregate hospital visits decrease by 16.3 days. More generally, for every $1 increase in pharmaceutical innovation hospital-care expenditures decrease by $3.65 (see also [5]). In another study, [6] finds that each new drug that is approved saves over 11,000 life-years, which equates to a social rate of return on innovation of approximately 40%. Additionally, [7] shows that, in France, pharmaceutical innovation is responsible for about 20% of the increase in longevity that occurred during the early 2000s. Consistent with earlier work that shows that innovation reduced hospital days, this latter study also highlights the association between innovation and a reduction in hospital visits. These results indicate that pharmaceutical innovation provides macro-level benefits to society.

In another stream of literature, researchers have identified more micro-level benefits associated with pharmaceutical innovation. For instance, a 2001 report from the Pharmaceutical Research and Manufacturers of America suggests that, since 1920, innovations in the pharmaceutical industry, such as antibiotics and vaccines, have nearly eradicated diseases like polio, syphilis, and diphtheria. Similarly, mortality rates associated with influenza and pneumonia have decreased by nearly 85% during the same period. Another study in [8] shows that pharmaceutical innovations, such as H2 blockers, proton pump inhibitors, and bronchodilators, have lowered death rates from ulcers, emphysema, and heart attacks by 60%, 31%, and 55%, respectively. Examining general longevity, [9] documents that innovations in cancer research have increased the life expectancy of the entire population–not just those diagnosed with cancer–by nearly 9%.

Despite these empirical results that highlight the societal benefits associated with pharmaceutical innovation, another branch of research suggests that the level of innovation in the pharmaceutical industry is decreasing substantially. [10] documents that, since the 1950s, the number of new and approved pharmaceuticals has been cut in half every nine years. For example, during the 1950s, every $1 billion in pharmaceutical spending resulted in the production of 40 new drugs. During the first half of the 2000s, every $1 billion in spending resulted in only 0.7 new drugs. More recently, [11] suggests that in the 1990s, about 37 new pharmaceuticals were approved per year; a number that has declined more than 40% during the 2000s.

Given the benefits of a robust pharmaceutical industry and the glaring decline in the industry rates of innovation, it becomes important for researchers to identify factors that might contribute to greater, or less, pharmaceutical innovation. This examination is motivated by the central and longstanding tenet in economics, that access to capital (through well-developed financial markets) is a necessary condition for innovation by firms ([12]). A variety of theoretical models have been developed to examine the role of intermediation and capital markets on growth and innovation. For example, [13] develop an endogenous growth model where financial intermediation promotes growth because it allows for a higher rate of return on capital, which can be used to fund costly financial structures. [14] creates a model where financial intermediation shifts the composition of savings towards capital, which promotes growth through investment. [15] constructs a model where banks ration credit to households while making it more available to firms, which leads to greater capital accumulation and economic growth. There are also several empirical studies that examine the relations between financial development, growth, and innovation. For instance, [16] show that robust financial markets promote growth through the allocation of capital to expanding industries over contracting industries–particularly for industries that rely more heavily on external financing. [17] finds that the development of capital markets leads to higher rates of innovation in technology by efficiently allocating capital to the most innovative firms. We extend this literature by examining the link between financial development and pharmaceutical innovation specifically.

Another stream of related literature examines the liberalization of financial markets in emerging economies. For instance, [18] finds that equity capital markets make it possible for firms to diversify financial risk. [19] shows that equity market liberalization improves capital allocation efficiency. [20] finds that equity market liberalizations lead to a 1% increase in average annual economic growth. [21] shows that financial liberalization has reduced stock market volatility and its sensitivity to information updates. [22] finds that banking development plays a key role in technological progress, and that banking deregulation significantly benefited the quality and quantity of innovative activities. [23] provides evidence that the banking environment affects the level and risk of innovation in private firms, which affects economic growth. [24] finds a negative relationship between bank distress and the level, quality, and trajectory of firm-level innovation during the great depression. [25] documents that competition between banks affects the innovation of public and private firms and that local credit allows small, innovative firms to avoid being acquired by public firms. [26] finds that credit markets foster growth in industries that rely on external financing for physical capital accumulation and play a key role in supporting economic growth. [27] examines the relation between banking and innovation in the Chinese credit market and find that long-term bank lending positively influences innovation, while short-term bank lending discourages innovation in certain industries (See also [28–44], among many others). We contribute to this research on the impact of financial markets on innovation by examining how cross-country differences in capital markets influence the innovation of new pharmaceuticals.

Understanding how financial markets influence pharmaceutical innovation is also helpful in recognizing the broader relationship between innovation and financial intermediation. For example, [45] explores the tension between firms going public or staying private and conclude that the incentives to innovate drive the decision. [46] finds that stronger shareholder protections, and access to stock market financing, leads to higher long-run rates of innovation. Furthermore, the authors show that credit market development only has a modest impact on fixed investment and no impact on research and development. More recently, however, [47] developed a model where financial intermediaries are the mechanism that assists innovation through entrepreneurial capital.

The objective of our study is to explore the possibility that better functioning capital markets can heighten innovation in the pharmaceutical industry. This objective has broad implications given the observed reduction in pharmaceutical innovation rates over the last 70 years. We gather data from a broad sample of countries from 1989 to 2016 and then, following prior research ([48]), we examine the following two measures of financial development. First, we obtain the amount of credit provided by banks and other financial institutions (relative to GDP) in a particular country. We denote this first measure of financial development as credit market development. Second, we gather data on liquidity in equity markets across countries as well as the market capitalization of all publicly traded firms in a particular country. We denote this second measure of financial development as equity market development. To approximate pharmaceutical innovation, we gather the dollar amount of pharmaceutical exports for each country each year. The idea behind the use of exports is based on [17], who use high-tech exports as a way to proxy for technological innovation within countries. We then conduct a series of univariate and multivariate tests that examine the association between pharmaceutical innovation and financial development. Our results show a remarkably strong relationship between pharmaceutical exports and credit market development. We also find that equity market development is associated with higher levels of pharmaceutical innovation–although to a much lesser extent. However, finding a correlation between our measures of financial development and pharmaceutical exports is not tantamount to documenting causality.

To draw stronger causal inferences, we use two approaches. First, we follow [49, 50], and use the legal origin of a country as an instrument for financial market development. We contend that legal origin serves as a valid instrument since it is related to financial market development, but unrelated to conditional current pharmaceutical innovation, as the legal origin of a particular country was established through occupation or colonization that occurred centuries before the sample period. Therefore, we use an indicator variable capturing whether a particular country has historically adopted an English legal origin and zero otherwise. Using a common two-stage least squares (2SLS) approach, we use the predicted values of credit and equity market development as the independent (exogenous) variable of interest. Results show that, when accounting for the potential endogeneity in our initial set of tests, the predicted values of both credit and equity market development are, again, directly associated with measures of pharmaceutical innovation. These results begin to allow us to make stronger causal inferences.

As a second method to draw stronger causal inferences, we use the adoption of the Euro as a natural experiment. Prior research shows that the implementation of the Euro resulted in a positive shock to both credit market and equity market development (see e.g., [51–53]). To the extent that this shock to financial development is exogenous in the framework of our study, the adoption of the Euro provides an additional way to identify a causal link between financial development and pharmaceutical innovation. Difference-in-difference tests show that, during the post-adoption period, pharmaceutical innovation increased in countries that adopted the Euro compared to countries that did not adopt the Euro. Combined with our instrumental variable analysis, these findings suggest that causation seems to run from credit and equity market development to pharmaceutical innovation instead of the other way around.

Our findings have broad implications for the existing literature and our understanding of the determinants of pharmaceutical innovation. [12]’s seminal work contends that financial development leads to economic growth because well-functioning financial markets can allocate capital efficiently and contribute to innovation and productivity. In a simple endogenous growth model, [54] shows that the aggregate production function that leads to higher economic output is driven by higher rates of capital investment. When financial markets properly allocate the flow of gross savings to gross investment, capital stock–and subsequently innovation–increases. Empirical research seems to support this argument (see e.g., [48, 50, 55–61]). In the framework of our study, countries with better-developed capital markets tend to exhibit greater pharmaceutical innovation and production. Existing research shows that pharmaceutical innovation can have a profound impact on a society’s standard of living (see e.g., [4–6, 62, 63]) and, therefore, the promotion of better-developed capital markets might assist in the innovative activities of the pharmaceutical industry.

## 2. Data description

The data used throughout the analysis come from two primary sources. From the World Bank (World Development Indicators Database), we gather macroeconomic information including GDP per capita (GDP), total exports and imports, gross savings, government expenditures, and capital formation. At the following URL, https://databank.worldbank.org/source/world-development-indicators, economic data can be obtained for all countries. Downloading the data from the WDI database is straightforward. The research can select all countries and specify the time period, which for our study extends from 1989 to 2016. In addition, under the series tab, the variables described above can be selected. From this first data source, we also gather domestic credit provided by banks and other financial institutions as well as stock market trading volumes and the market capitalization of all domestically traded securities. These variables are used as measures of credit market and equity market development. As a second data source, we gather pharmaceutical exports from the UN Comtrade Database. At the following URL, comtrade.un.org/data/, export data can be obtained by including the years 1989 to 2016. By default, the UN comtrade includes all reporting countries and all trade partners at the annual. Additionally, by default, all trade flows are included when pulling the data. After including the years, the data can be downloaded into a workable.csv file. We note that the pharmaceutical export data is not available before the late 1980s, and therefore, we restrict our sample from 1989 to 2016. Since not all of the countries have available data in each year of the sample period, we carefully denote the number of observations that are included in each of the tests. Our entire dataset includes data from the universe of countries on the World Development Indicators (WDI) Database, which equates to about 6,000 country-year observations.

We examine the following variables in the empirical analysis: *Pharma* is the number of pharmaceutical exports in billions of U.S. dollars. *Pharma/GDP* is the amount of pharmaceutical exports to GDP. *Pharma/Ex* is the amount of pharmaceutical exports to total exports. *Banks* is the domestic credit offered by banks as a percent of GDP. *Finance* is the domestic credit offered by the financial sector as a percent of GDP. *Private* is the total domestic credit offered by various financial institutions to the private sector as a percent of GDP. *Turn* is the amount of share turnover–or the ratio of dollar trading volume on a country’s stock exchanges relative to the total market capitalization of all domestic, publicly traded stocks. *Vol/GDP* is the ratio of dollar volume on all domestic stock exchanges to GDP. *MCAP/GDP* is the ratio of the market capitalization of all domestic stocks that are publicly traded to GDP. *GDP/Capita* is GDP per capita. *NetEx* is the difference between imports and exports (in billions). *Save/GDP* is the amount of gross savings relative to GDP. *GovSpend* is the amount of national expenditures in billions of U.S. dollars. *CapForm* is the amount of capital formation in billions of U.S. dollars.

In Table 1, we report the summary statistics that describe the cross-country sample. All variables are calculated at the country/year level. The average amount spent on pharmaceutical exports is $1.8612 billion, which represents 0.4422 percent of GDP and 0.8898 percent of all exports. There is, however, significant variation in the amount of pharmaceutical exports across countries, as the standard deviation is $7.2692 billion. The average amount of credit offered by banks, as a percent of GDP, is 41.95. The average amount of credit offered by the finance sector, as a percent of GDP, is 44.14 while the total amount of credit offered to the private sector (as a percent of GDP) is 58.07, respectively. The average share turnover is 48.38, with a standard deviation of 76.51. The average volume to GDP and market capitalization to GDP are 30.90 and 60.51, respectively. While the mean per capita GDP is $11,335.72, the standard deviation is $18,738.75. The average difference between imports and exports is $1.24 billion. The average amount of gross savings, as a percent of GDP, is 17.80. The average amount of national expenditures is $280.23 billion, and the average amount of capital formation is $68.82 billion.

**Table 1:** Summary statistics.

Panel A. Pharmaceutical innovation								
	N	Mean	Std. Dev.	Minimum	25^th^	Median	75^th^	Maximum
	[1]	[2]	[3]	[4]	[5]	[6]	[7]	[8]
*Pharma*	3,845	1.8612	7.2692	0.0000	0.0001	0.0251	0.2885	84.4700
*Pharma/GDP*	3,424	0.4422	1.2420	0.0000	0.0091	0.0583	0.3258	14.7800
*Pharma/Ex*	3,370	0.8898	1.7875	0.0000	0.0323	0.1895	0.9189	15.3200
Panel B. Credit market development								
*Banks*	4,517	41.95	38.62	0.00	14.24	29.65	56.03	312.12
*Finance*	4,506	44.14	41.16	0.00	14.76	30.67	58.28	312.12
*Private*	4,498	58.07	62.21	-114.69	21.42	42.85	75.29	2,066.18
Panel C. Equity market development								
*Turn*	1,703	48.38	76.51	0.02	7.97	28.09	63.57	1,721.54
*Vol/GDP*	1,972	30.90	64.14	0.00	1.70	8.38	34.98	952.67
*MCAP/GDP*	1,743	60.51	95.92	0.01	18.47	37.00	73.37	1,254.47
Panel D. Controls								
*GDP/Capita*	5,510	11,335.72	18,738.75	65.01	913.59	3,243.69	13,882.86	192,989.19
*NetEx*	5,110	1.24	41.07	-770.94	-1.43	-0.20	0.80	385.50
*Save/GDP*	4,696	17.80	18.49	-167.61	8.91	19.26	27.01	83.72
*GovSpend*	4,683	280.23	1,168.49	0.07	4.95	19.32	118.52	19,145.70
*CapForm*	4,719	68.82	293.20	-0.03	0.98	3.97	29.05	5,023.46

The table reports statistics that describe the sample of 240 countries over the period 1989 to 2016. Panel A reports statistics on the following measures of pharmaceutical innovation: *Pharma* is the number of pharmaceutical exports in billions of U.S. dollars. *Pharma/GDP* is the amount of pharmaceutical exports scaled by GDP. *Pharma/Ex* is the amount of pharmaceutical exports scaled by total exports. Panel B reports statistics on the following measures of credit market development: *Banks* is the domestic credit offered by banks as a percent of GDP. *Finance* is the domestic credit offered by the financial sector as a percent of GDP. *Private* is the domestic credit offered by the private sector as a percent of GDP. Panel C reports statistics on the following measures of equity market development: *Turn* is the amount of share turnover–or the ratio of dollar trading volume on a country’s stock exchanges relative to total market capitalization of all domestic, publicly traded stocks. *Vol/GDP* is the ratio of Volume to GDP. *MCAP/GDP* is the ratio of market capitalization of all domestic stocks to GDP. Panel D reports statistics for the following control measures: *GDP/Capita* is GDP per capita. *NetEx* is the difference between imports and exports (in billions). *Save/GDP* is the amount of gross savings relative to GDP. *GovSpend* is the amount of national expenditures in billions of U.S. dollars. *CapForm* is the amount of capital formation in billions of U.S. dollars.

In Table 2, we report the correlation matrix for the cross-country sample. As expected, the measures of pharmaceutical innovation are positively correlated. In fact, the correlation coefficient between is *Pharma/GDP* and *Pharma/Ex* is 0.9267. Perhaps more interestingly, all measures of credit market development, i.e., *Banks*, *Finance*, and *Private*, are positively correlated with pharmaceutical innovation. For example, the correlation coefficient between *Banks* and *Pharma* is 0.3385. Similar correlation coefficients are found between *Finance* and *Pharma* (0.3215) and *Private* and *Pharma* (0.3668). Furthermore, we find that all three measures of equity market development are positively correlated with pharmaceutical innovation. For instance, the correlation between share turnover and total pharmaceutical exports is 17.48 percent. In addition, the correlation coefficients between *Vol/GDP* and *Pharma* and *MCAP/GDP* and *Pharma* are 0.1722 and 0.0788, respectively. These preliminary results suggest that credit market development and equity market development are positively correlated with pharmaceutical innovation, with the former appearing to be more significant.

**Table 2:** Correlation matrix.

	*Pharma*	*Pharma/GDP*	*Pharma/Ex*	*Banks*	*Finance*	*Private*	*Turn*	*Vol/GDP*	*MCAP/GDP*	*GDP/Capita*	*NetEx*	*Save/ GDP*	*Gov Spend*	*CapForm*
	[1]	[2]	[3]	[4]	[5]	[6]	[7]	[8]	[9]	[10]	[11]	[12]	[13]	[14]
*Pharma*	1.0000	0.6319	0.6958	0.3385	0.3215	0.3668	0.1748	0.1722	0.0788	0.4546	0.3291	0.1138	0.4075	0.2648
*Pharma/GDP*		1.0000	0.9267	0.3370	0.3162	0.3398	-0.0149	0.0710	0.0752	0.4119	0.1497	0.1395	0.0474	0.0213
*Pharma/Ex*			1.0000	0.3969	0.3728	0.3905	0.0376	0.0924	0.0551	0.4558	0.1082	0.0814	0.1422	0.0764
*Banks*				1.0000	0.9657	0.7197	0.1757	0.4295	0.4452	0.6498	0.1094	0.2900	0.2292	0.2577
*Finance*					1.0000	0.7592	0.2280	0.4924	0.4372	0.6629	-0.0580	0.2768	0.4294	0.4176
*Private*						1.0000	0.2222	0.3897	0.3156	0.5248	-0.0592	0.1546	0.4386	0.4214
*Turn*							1.0000	0.3450	0.0397	0.1219	-0.0480	0.0563	0.3157	0.3350
*Vol/GDP*								1.0000	0.8076	0.2756	-0.1268	0.1711	0.3638	0.3479
*MCAP/GDP*									1.0000	0.2787	-0.0145	0.2175	0.0817	0.0700
*GDP/Capita*										1.0000	0.0390	0.3862	0.2896	0.2496
*NetEx*											1.0000	0.1575	-0.4871	-0.2752
*Save/GDP*												1.0000	0.0901	0.1250
*GovSpend*													1.0000	0.9378
*CapForm*														1.0000

The table reports correlation coefficients for the variables used in the analysis. *Pharma* is the number of pharmaceutical exports in billions of U.S. dollars. *Pharma/GDP* is the amount of pharmaceutical exports scaled by GDP. *Pharma/Ex* is the amount of pharmaceutical exports scaled by total exports. *Banks* is the domestic credit offered by banks as a percent of GDP. *Finance* is the domestic credit offered by the financial sector as a percent of GDP. *Private* is the domestic credit offered by the private sector as a percent of GDP. *Turn* is the amount of share turnover–or the ratio of dollar trading volume on a country’s stock exchanges relative to the total market capitalization of all domestic, publicly traded stocks. *Vol/GDP* is the ratio of Volume to GDP. *MCAP/GDP* is the ratio of market capitalization to GDP. *GDP/Capita* is GDP per capita. *NetEx* is the difference between imports and exports (in billions). *Save/GDP* is the amount of gross savings relative to GDP. *GovSpend* is the amount of national expenditures in billions of U.S. dollars. *CapForm* is the amount of capital formation in billions of U.S. dollars.

## 3. Empirical results

In this section, we provide a detailed discussion of our empirical findings. First, we examine whether credit market development impacts pharmaceutical innovation. Second, we analyze whether equity market development affects pharmaceutical innovation. Third, we use the adoption of the Euro as a shock to financial market development and examine the change in pharmaceutical innovation for the adopting countries vis-à-vis the non-adopting countries.

### 3.1. Credit markets and pharmaceutical innovation

In this subsection, we analyze the effect of credit market development on pharmaceutical innovation. To do so, we estimate the following regression equation specifications on annual cross-country data:

$${Ln(PharmaInnov}_{i,t})=\alpha +\gamma_{t}+\beta_{1}{Ln(Credit}_{i,t})+\beta_{2}{Ln(GDP/Capita}_{i,t})+\beta_{3}{NetEx}_{i,t}+\beta_{4}{Ln(Save/GDP}_{i,t})+\beta_{5}{Ln(GovSpend}_{i,t})+\beta_{6}{Ln(CapForm}_{i,t})+\varepsilon_{i,t},$$ (1)

where the dependent variable is the natural log of one of three measures of pharmaceutical innovation: *Pharma*, *Pharma/GDP*, and *Pharma/Ex*. The independent variable of interest is the natural log of one of three measures of credit market development: *Banks*, *Finance*, and *Private*. We include year fixed effects, *γ*_*t*_, and control for several other country-specific variables that have previously been defined. We report the estimated coefficients from Eq (1) in Table 3 with *t*-statistics in parentheses obtained from robust standard errors.

**Table 3:** Pharmaceutical innovation and credit market development.

	Ln(Pharma)			Ln(Pharma/GDP)			Ln(Pharma/Ex)		
	[1]	[2]	[3]	[4]	[5]	[6]	[7]	[8]	[9]
*Ln(Banks)*	0.6346***			0.6569***			0.5071***		
	(4.69)			(4.73)			(4.54)		
*Ln(Finance)*		0.6066***			0.6317***			0.5207***	
		(4.89)			(4.94)			(4.85)	
*Ln(Private)*			0.6111***			0.6337***			0.4888***
			(4.65)			(4.69)			(4.49)
*Ln(GDP/Capita)*	0.6235***	0.6474***	0.6325***	0.5932***	0.6166***	0.6021***	0.4394***	0.4434***	0.4465***
	(10.51)	(12.53)	(10.89)	(9.78)	(11.67)	(10.13)	(8.32)	(9.28)	(8.60)
*NetEx*	-0.0029***	-0.0024**	-0.0028***	-0.0036***	-0.0031***	-0.0036***	-0.0058***	-0.0053***	-0.0058***
	(-3.15)	(-2.56)	(-3.06)	(-3.88)	(-3.26)	(-3.78)	(-6.44)	(-5.93)	(-6.31)
*Ln(Save/GDP)*	-0.3606***	-0.3334***	-0.3630***	-0.4743***	-0.4449***	-0.4766***	-0.6893***	-0.6604***	-0.6911***
	(-4.54)	(-4.17)	(-4.58)	(-5.72)	(-5.35)	(-5.76)	(-7.60)	(-7.32)	(-7.63)
*Ln(GovSpend)*	2.1465***	2.0254***	2.1350***	1.0495***	0.9232***	1.0378***	1.2862***	1.1878***	1.2776***
	(10.37)	(10.00)	(10.35)	(4.98)	(4.49)	(4.94)	(5.90)	(5.58)	(5.88)
*Ln(CapForm)*	-0.8923***	-0.7891***	-0.8820***	-0.7883***	-0.6813***	-0.7780***	-0.8367***	-0.7551***	-0.8290***
	(-4.31)	(-3.91)	(-4.27)	(-3.73)	(-3.32)	(-3.70)	(-3.84)	(-3.56)	(-3.81)
*Constant*	-21.2922***	-21.0599***	-21.2470***	-20.7829***	-20.5320***	-20.7355***	-22.0365***	-21.8268***	-22.0017***
	(-30.39)	(-29.71)	(-30.21)	(-29.48)	(-28.82)	(-29.30)	(-32.04)	(-31.56)	(-31.91)
Adjusted R^2^	0.7495	0.7500	0.7485	0.4008	0.3996	0.3985	0.3943	0.3970	0.3928
Robust SEs	Yes	Yes	Yes	Yes	Yes	Yes	Yes	Yes	Yes
N	2,654	2,630	2,652	2,654	2,630	2,652	2,654	2,630	2,652

The table reports the results from estimating the following equation on annual cross-country data:

${Ln(PharmaInnov}_{i,t})=\alpha +\gamma_{t}+\beta_{1}{Ln(Credit}_{i,t})+\beta_{2}{Ln(GDP/Capita}_{i,t})+\beta_{3}{NetEx}_{i,t}+\beta_{4}{Ln(Save/GDP}_{i,t})+\beta_{5}{Ln(GovSpend}_{i,t})+\beta_{6}{Ln(CapForm}_{i,t})+\varepsilon_{i,t},$

where the dependent variable is the natural log of one of three measures of pharmaceutical innovation: *Pharma* is the number of pharmaceutical exports (in $billions), *Pharma/GDP* is the amount of pharmaceutical exports relative to GDP, and *Pharma/Ex* is the amount of pharmaceutical exports relative to total exports. The independent variable of interest is the natural log of one of three measures of credit market development: *Banks* is the percent of domestic credit offered by banks relative to GDP, *Finance* is the amount of domestic credit offered by the finance sector as a percent of GDP, and *Private* is the amount of domestic credit offered by the private sector scaled by GDP. The control variables include the following: *Ln(GDP/Capita)* is the natural log of GDP per capita. *NetEx* is the difference between exports and imports (in billions of U.S. dollars). *Ln(Save/GDP)* is the natural log of the ratio of gross savings to GDP. *Ln(GovSpend)* is the natural log of the national expenditures (in billions of U.S. dollars). *Ln(CapForm)* is the natural log of capital formation in billions of U.S. dollars. We include year fixed effects and report *t*-statistics in parentheses obtained from robust standard errors.

*, **, and *** denote statistical significance at the 0.10, 0.05, and 0.01 levels, respectively.

In the first three columns of Table 3, we report the results from estimating Eq (1) inserting the natural log of the total amount of pharmaceutical exports as the dependent variable. We find that a one percent increase in the amount of credit offered by banks, relative to GDP, is associated with a 0.6346 percent increase in total pharmaceutical exports. Additionally, a one percent increase in the amount of credit offered by the finance sector (to the private sector), as a percent of GDP, is associated with a 0.6066 (0.6111) percent increase in pharmaceutical exports. These results suggest a positive and significant relation between credit market development and pharmaceutical innovation.

In columns [4] through [6] of Table 3, we tabulate the results from estimating Eq (1) inputting the natural log of the ratio of pharmaceutical exports to GDP as the dependent variable. Other factors held constant; we find that a percent increase in the amount of credit offered by banks is associated with a 0.6569 percent increase in the ratio of pharmaceutical exports to GDP. Furthermore, a one percent increase in the amount of credit offered by the finance sector (to the private sector) is associated with a 0.6317 (0.6337) percent increase in the amount of pharmaceutical exports to GDP. Again, these results indicate a positive association between credit market development and pharmaceutical innovation.

In the final three columns of Table 3, we present the results from estimating Eq (1) when the natural log of pharmaceutical exports to total exports is the dependent variable. We find that a percent increase in credit offered by banks is associated with a 0.5071 percent increase in pharmaceutical exports to total exports. In addition, we find that a one percent increase in credit offered by the finance sector (private sector) is associated with a 0.5207 (0.4888) percent increase in pharmaceutical exports to total exports. These results hold after controlling for time-series variation and country-level characteristics. The results in Table 3 provide consistent evidence that credit market development is positively related to pharmaceutical innovation.

### 3.2. Equity market development and pharmaceutical innovation

In our next set of tests, we examine the effect of equity market development on pharmaceutical innovation. Similar to before, we estimate specifications of the following regression equation on yearly cross-country data:

$${Ln(PharmaInnov}_{i,t})=\alpha +\gamma_{t}+\beta_{1}{Ln(Equity}_{i,t})+\beta_{2}{Ln(GDP/Capita}_{i,t})+\beta_{3}{NetEx}_{i,t}+\beta_{4}{Ln(Save/GDP}_{i,t})+\beta_{5}{Ln(GovSpend}_{i,t})+\beta_{6}{Ln(CapForm}_{i,t})+\varepsilon_{i,t},$$ (2)

where the dependent variable is the natural log of one of three measures of pharmaceutical innovation: *Pharma*, *Pharma/GDP*, *Pharma/Ex*. The independent variable of interest is the natural log of one of three measures of economic development: *Turn*, *Vol/GDP*, and *MCAP/GDP*. We include year fixed effects, *γ*_*t*_, and various country-specific control variables. These variables have been defined previously. We report the results from estimating Eq (2) in Table 4, with *t*-statistics in parentheses obtained from robust standard errors.

**Table 4:** Pharmaceutical innovation and equity market development.

	Ln(Pharma)			Ln(Pharma/GDP)			Ln(Pharma/Ex)		
	[1]	[2]	[3]	[4]	[5]	[6]	[7]	[8]	[9]
*Ln(Turn)*	0.1377***			0.1391***			0.0745*		
	(3.37)			(3.36)			(1.92)		
*Ln(Vol/GDP)*		0.1092***			0.1050***			0.0069	
		(4.28)			(4.08)			(0.27)	
*Ln(MCAP/GDP)*			0.0535			0.0383			-0.1132**
			(1.29)			(0.92)			(-2.54)
*Ln(GDP/Capita)*	0.9086***	0.8356***	0.9036***	0.8892***	0.8181***	0.8886***	0.6973***	0.6653***	0.7407***
	(19.33)	(18.44)	(18.02)	(18.71)	(17.81)	(17.53)	(14.97)	(14.52)	(14.81)
*NetEx*	-1.4E-5	-0.0009	-0.0004	-0.0005	-0.0015	-0.0009	-0.0025***	-0.0035***	-0.0032***
	(-0.01)	(-0.92)	(-0.42)	(-0.49)	(-1.45)	(-0.93)	(-2.66)	(-3.55)	(-3.37)
*Ln(Saving)*	-0.3506**	-0.4332***	-0.3412**	-0.4931***	-0.5704***	-0.4780***	-0.8335***	-0.8678***	-0.7759***
	(-2.26)	(-2.99)	(-2.28)	(-3.06)	(-3.76)	(-3.09)	(-4.88)	(-5.33)	(-5.00)
*Ln(GovSpend)*	1.3932***	1.4285***	1.3579***	0.3227	0.3555	0.2759	0.8103***	0.7615**	0.6477**
	(4.64)	(4.58)	(4.23)	(1.05)	(1.11)	(0.84)	(2.72)	(2.43)	(2.18)
*Ln(CapForm)*	-0.4657	-0.5000	-0.3562	-0.3810	-0.4111	-0.2575	-0.5674*	-0.5004	-0.3497
	(-1.54)	(-1.58)	(-1.10)	(-1.23)	(-1.26)	(-0.77)	(-1.90)	(-1.57)	(-1.17)
*Constant*	-12.8991***	-11.8007***	-14.2963***	-12.5394***	-11.5020***	-13.9760***	-16.6728***	-16.3864***	-17.5785***
	(-10.71)	(-11.68)	(-14.23)	(-10.31)	(-11.23)	(-13.63)	(-14.62)	(16.79)	(-18.35)
Adjusted R^2^	0.6790	0.6572	0.6783	0.3147	0.2841	0.3113	0.3293	0.2916	0.3266
Robust SEs	Yes	Yes	Yes	Yes	Yes	Yes	Yes	Yes	Yes
N	1,395	1,625	1,451	1,395	1,625	1,451	1,395	1,625	1,451

The table reports the results from estimating the following equation used an unbalanced panel of countries and years.

${Ln(PharmaInnov}_{i,t})=\alpha +\gamma_{t}+\beta_{1}{Ln(Equity}_{i,t})+\beta_{2}{Ln(GDP/Capita}_{i,t})+\beta_{3}{NetEx}_{i,t}+\beta_{4}{Ln(Save/GDP}_{i,t})+\beta_{5}{Ln(GovSpend}_{i,t})+\beta_{6}{Ln(CapForm}_{i,t})+\varepsilon_{i,t},$

where the dependent variable is the natural log of one of three measures of pharmaceutical innovation: *Pharma* is the number of pharmaceutical exports (in $billions), *Pharma/GDP* is the amount of pharmaceutical exports relative to GDP, and *Pharma/Ex* is the amount of pharmaceutical exports relative to total exports. The independent variable of interest is the natural log of one of three measures of economic development: *Turn* is equal to total dollar volume (on all domestic stock exchanges) scaled by market capitalization, *Vol/GDP* is the ratio of total dollar volume to GDP, and *MCAP/GDP* is the total market capitalization of all publicly traded stocks in the domestic country as a percent of GDP. The control variables include the following: *Ln(GDP/Capita)* is the natural log of GDP per capita. *NetEx* is the difference between exports and imports (in billions of U.S. dollars). *Ln(Save/GDP)* is the natural log of the ratio of gross savings to GDP. *Ln(GovSpend)* is the natural log of the national expenditures (in billions of U.S. dollars). *Ln(CapForm)* is the natural log of capital formation in billions of U.S. dollars. We include year fixed effects and report *t*-statistics in parentheses obtained from robust standard errors.

*, **, and *** denote statistical significance at the 0.10, 0.05, and 0.01 levels, respectively.

In columns [1] through [3] of Table 4, we report the results from estimating Eq (2) when total pharmaceutical exports is the measure of innovation. Here, we find that a one percent increase in stock turnover is associated with a 0.1377 percent increase in total pharmaceutical exports. Likewise, a one percent increase in the ratio of trading volume to GDP is associated with a 0.1092 percent increase in total pharmaceutical exports. We fail to find a significant relation between the ratio of market capitalization to GDP and total pharmaceutical exports. Specifically, in column [3] we find a *β*_1_ coefficient equal to 0.0535, which is not reliably different from zero. Therefore, the results suggest that while stock market liquidity is directly associated with pharmaceutical innovation, the size of the stock market is not.

We re-estimate Eq (2) inserting the ratio of total pharmaceutical exports to GDP as the dependent variable, and report the results in columns [4] through [6] of Table 4. Similar to before, we find that a percent increase in share turnover (volume/GDP) is associated with a 0.1391 (0.1050) percent increase in the ratio of pharmaceutical exports to GDP. These results are robust to year fixed effects and various country-specific control measures. However, we do not find a significant relation between the ratio of market capitalization to GDP and the ratio of pharmaceutical exports to GDP.

In the final three columns of Table 4, we report the results from estimating Eq (2) inputting the ratio of pharmaceutical exports to total exports as the dependent variable. We find that a one percent increase in share turnover is associated with a 0.0745 percent increase in the ratio of pharmaceutical exports to total exports, albeit the result is only significant at the 0.10 level. Interestingly, we find a negative and significant coefficient on *β*_1_ in the final column, indicating an inverse relation between the ratio of market capitalization to GDP and the percentage of total exports that are pharmaceutical. Specifically, a one percent increase in the ratio of market capitalization to GDP is associated with a 0.1132 percent *decrease* in pharmaceutical exports to total exports.

Thus, the results in Table 4 seem to suggest a positive relation between equity market liquidity and pharmaceutical innovation. However, we do not find that the size of the equity market–as measured by market capitalization–influences the level of innovation. Thus, the relation between both pharmaceutical innovation and equity market development depends on the measure of development.

### 3.3. Credit and equity market development and pharmaceutical innovation

In this subsection, we analyze whether the positive relation between credit market development and pharmaceutical innovation supersedes the positive relation between equity market development and pharmaceutical innovation, or vice-versa. Therefore, we estimate the following regression equation on a panel of cross-country data, which combines terms from Eqs (1) and (2):

$${Ln(PharmaInnov}_{i,t})=\alpha +\gamma_{t}+\beta_{1}{Ln(Credit}_{i,t})+\beta_{2}{Ln(Turn}_{i,t})+\beta_{3}{Ln(GDP/Capita}_{i,t})+\beta_{4}{NetEx}_{i,t}+\beta_{5}{Ln(Save/GDP}_{i,t})+\beta_{6}{Ln(GovSpend}_{i,t})+\beta_{7}{Ln(CapForm}_{i,t})+\varepsilon_{i,t},$$ (3)

where the dependent variable is the natural log of one of three measures of pharmaceutical innovation: *Pharma*, *Pharma/GDP*, *Pharma/Ex*. The independent variables have been defined previously. In unreported tests, we find that the correlations between turnover, volume, and market capitalization are significant enough to produce variance inflation factors that signal potential multicollinearity. For this reason, we only report the estimated coefficients from Eq (3) when turnover is used to proxy equity market development. However, we note that the results are similar when volume to GDP or market capitalization to GDP are substituted as the measure of equity market development. We suppress the estimated coefficients on the control variables in order to conserve space and only report the results on the credit and equity market development estimates. The results of estimating Eq (3) are found in Table 5, with *t*-statistics in parentheses obtained from robust standard errors.

**Table 5:** Pharmaceutical innovation and both credit and equity market development.

	Ln(Pharma)			Ln(Pharma/GDP)			Ln(Pharma/Ex)		
	[1]	[2]	[3]	[4]	[5]	[6]	[7]	[8]	[9]
*Ln(Banks)*	0.5125***			0.5454***			0.4123***		
	(3.99)			(4.11)			(3.46)		
*Ln(Finance)*		0.5513***			0.5734***			0.4801***	
		(4.43)			(4.49)			(4.01)	
*Ln(Private)*			0.4128***			0.4458***			0.3308***
			(3.40)			(3.55)			(2.92)
*Ln(Turn)*	0.0571	0.0896**	0.0737	0.0546	0.0900**	0.0716	0.0056	0.0296	0.0191
	(1.24)	(2.06)	(1.63)	(1.16)	(2.02)	(1.55)	(0.12)	(0.66)	(0.42)
Adjusted R^2^	0.7027	0.7056	0.6998	0.3638	0.3690	0.3574	0.3586	0.3646	0.3541
Controls	Yes	Yes	Yes	Yes	Yes	Yes	Yes	Yes	Yes
Robust SEs	Yes	Yes	Yes	Yes	Yes	Yes	Yes	Yes	Yes
N	1,240	1,235	1,240	1,240	1,235	1,240	1,240	1,235	1,240

The table reports the results from estimating the following equation using an unbalanced panel of countries and years.

${Ln(PharmaInnov}_{i,t})=\alpha +\gamma_{t}+\beta_{1}{Ln(Credit}_{i,t})+\beta_{2}{Ln(Turn}_{i,t})+\beta_{3}{Ln(GDP/Capita}_{i,t})+\beta_{4}{NetEx}_{i,t}+\beta_{5}{Ln(Save/GDP}_{i,t})+\beta_{6}{Ln(GovSpend}_{i,t})+\beta_{7}{Ln(CapForm}_{i,t})+\varepsilon_{i,t},$

where the dependent variable is the natural log of one of three measures of pharmaceutical innovation: *Pharma* is the number of pharmaceutical exports (in $billions), *Pharma/GDP* is the amount of pharmaceutical exports relative to GDP, and *Pharma/Ex* is the amount of pharmaceutical exports relative to total exports. The independent variable, *Ln(Credit)*, is the natural log of one of three measures of credit market development: *Banks* is the percent of domestic credit offered by banks relative to GDP, *Finance* is the amount of domestic credit offered by the finance sector as a percent of GDP, and *Private* is the amount of domestic credit offered by the private sector scaled by GDP. *Ln(Turn)* is the natural log of total dollar volume (on all domestic stock exchanges) scaled by market capitalization. The control variables include the following: *Ln(GDP/Capita)* is the natural log of GDP per capita. *NetEx* is the difference between exports and imports (in billions of U.S. dollars). *Ln(Save/GDP)* is the natural log of the ratio of gross savings to GDP. *Ln(GovSpend)* is the natural log of the national expenditures (in billions of U.S. dollars). *Ln(CapForm)* is the natural log of capital formation in billions of U.S. dollars. We include year fixed effects and report *t*-statistics in parentheses obtained from robust standard errors.

*, **, and *** denote statistical significance at the 0.10, 0.05, and 0.01 levels, respectively.

In the first three columns of Table 5, we report the results from the simultaneous estimation of credit and equity market development on total pharmaceutical exports. In the first column, we find that a one percent increase in bank credit leads to a 0.5125 percent increase in total pharmaceutical exports, holding constant the level of equity market development in the same period. In contrast, we do not find that equity market development significantly affects total pharmaceutical exports, holding constant the amount of credit offered by banks. In the second column, we find that a one percent increase in financial credit is associated with a 0.5513 percent increase in total pharmaceutical exports, other factors held constant. We note that a percent increase in stock turnover leads to a 0.0896 percent increase in total pharmaceutical exports, holding constant credit market development. In the third column, we find that a one percent increase in the total amount of credit offered to the private sector is associated with a 0.4128 percent increase in total pharmaceutical exports, controlling for equity market development. We find that the effect of turnover on pharmaceutical innovation disappears after controlling for credit offered to the private sector.

The findings are similar in columns [4] through [9] of Table 5, when we estimate pharmaceutical innovation as a percentage of GDP or as a percentage of total exports. These results suggest that the link between credit market development and pharmaceutical innovation is stronger than that of equity market development and pharmaceutical innovation.

### 3.4. Accounting for potential endogeneity–Instrumental variable analysis

The results in Table 3 through 5 provide evidence of a strong, positive relation between credit market and equity market development and pharmaceutical innovation. To account for the potential endogeneity in our tests and to draw stronger causal inferences, we first attempt to identify a valid instrument for financial market development. Following [49, 50]), we use legal origin as an instrument. [49] finds that both equity market and credit market development are stronger in countries that have adopted English legal origin, or common law, where judges and legislators make laws that govern society. On the other hand, French, German, and Scandinavian legal origins are based more heavily on the civil law tradition. Given the evidence in [49, 64], this instrument is likely to be correlated with our measures of financial development. Additionally, since the legal origin of countries was adopted hundreds of years before our sample period, our instrument is likely uncorrelated with the error term in either Eq (4) or (5), thus satisfying the exclusion restriction. Further discussion of the validity of legal origin as an instrumental variable can be found in [65], where they examine the validity of legal origin as an exogenous instrument using an over-identifying restrictions test as well as additional instruments.

To implement our instrumental variable analysis, we estimate the following two equations using a 2SLS approach:

$${Ln(Credit\;or\;Equity}_{i,t})=\alpha +\gamma_{t}+\beta_{1}{Ln(English\;Origin}_{i})+\beta_{2}{Ln(GDP/Capita}_{i,t})+\beta_{3}{NetEx}_{i,t}+\beta_{4}{Ln(Save/GDP}_{i,t})+\beta_{5}{Ln(GovSpend}_{i,t})+\beta_{6}{Ln(CapForm}_{i,t})+\varepsilon_{i,t}$$ (4)

and

$${Ln(PharmaGDP}_{i,t})=\alpha +\gamma_{t}+\beta_{1}{Ln(Credi\hat{t\;or\;}Equity}_{i,t})+\beta_{2}{Ln(GDP/Capita}_{i,t})+\beta_{3}{NetEx}_{i,t}+\beta_{4}{Ln(Save/GDP}_{i,t})+\beta_{5}{Ln(GovSpend}_{i,t})+\beta_{6}{Ln(CapForm}_{i,t})+\varepsilon_{i,t}$$ (5)

where the dependent variable in the first stage (Eq (4)) is the natural log of one of six measures of credit or equity market development. The instrument in the first stage is the indicator variable capturing countries that historically adopted an English legal origin and zero otherwise. The dependent variable in the second stage (Eq (5)) is the natural log of *Pharma/GDP*, which is the amount of pharmaceutical exports relative to GDP. We note that we only tabulate the results using pharmaceutical exports relative to GDP as our dependent variable in the second stage for brevity. However, we are able to draw similar conclusions when we use the natural log of total pharmaceutical exports or the natural log of the ratio between pharmaceutical exports to total exports. The independent variable of interest in the second stage is the predicted values from the first-stage regression. The other control variables have previously been defined. We also include year fixed effects, *γ*_*t*_, in both stages of the 2SLS analysis. Again, for brevity, we suppress the coefficients on all the control variables but report the results for the variable of interest in both the first and the second-stage regressions.

We first estimate Eq (4) using one of three measures of credit market development (*Ln(Credit)*) as the dependent variable. We then estimate Eq (5) using the predicted values on credit market development in the first-stage regression as the independent variable of interest. The results of this 2SLS analysis are reported in Table 6. In columns [1] and [2], the potentially endogenous variable is *Ln(Banks*). Consistent with the results in La Porta et al. (1997), we find that the coefficient on the *English Origin* in the first-stage regression is positive and statistically significant. More importantly, we find that the predicted values on *Ln(Banks)* produces a positive and significant coefficient in the second-stage regression. In economic terms, a one percent increase in the predicted value of *Ln(Banks)* is associated with a 1.27% increase in pharmaceutical innovation. Qualitatively similar results are found in columns [3] and [4] and in columns [5] and [6] when using the other measures of credit market development. These results seem to suggest that, after accounting for potential endogeneity, credit market development influences the level of pharmaceutical innovation.

**Table 6:** Pharmaceutical innovation and credit market development–Instrumental variable analysis.

	1^st^ Stage	2^nd^ Stage	1^st^ Stage	2^nd^ Stage	1^st^ Stage	2^nd^ Stage
	[1]	[2]	[3]	[4]	[5]	[6]
*English Origin*	0.4821***		0.3603***		0.4701***	
	(11.72)		(8.57)		(11.35)	
*Ln(Banks)*		1.2737***				
		(5.11)				
*Ln(Finance)*				1.6664***		
				(4.79)		
*Ln(Private)*						1.3100***
						(5.09)
Adj. R^2^	0.5254	0.3727	0.4838	0.3526	0.5243	0.3693
Controls	Yes	Yes	Yes	Yes	Yes	Yes
N	2,654	2,654	2,630	2,630	2,652	2,652

The table reports the results from estimating the following equations on annual cross-country data using 2SLS:

${Ln(Credit}_{i,t})=\alpha +\gamma_{t}+\beta_{1}{English\;Origin}_{i}+\beta_{2}{Ln(GDP/Capita}_{i,t})+\beta_{3}{NetEx}_{i,t}+\beta_{4}{Ln(Save/GDP}_{i,t})+\beta_{5}{Ln(GovSpend}_{i,t})+\beta_{6}{Ln(CapForm}_{i,t})+\varepsilon_{i,t},$

and

${Ln(PharmaGDP}_{i,t})=\alpha +\gamma_{t}+\beta_{1}{Ln(C\hat{red}it}_{i,t})+\beta_{2}{Ln(GDP/Capita}_{i,t})+\beta_{3}{NetEx}_{i,t}+\beta_{4}{Ln(Save/GDP}_{i,t})+\beta_{5}{Ln(GovSpend}_{i,t})+\beta_{6}{Ln(CapForm}_{i,t})+\varepsilon_{i,t},$

where the dependent variable in the first stage is the natural log of one of three measures of credit market development. The instrument in the first stage is an indicator variable capturing countries that historically adopted an English Legal Origin (following La Porta et al. 1997)–zero otherwise. The dependent variable in the second stage is the natural log of *Pharma/GDP*, which is the amount of pharmaceutical exports relative to GDP. The independent variable of interest in the second stage is the predicted value on *Ln(Credit)*, which is the natural log of one of three measures of credit market development: *Banks* is the percent of domestic credit offered by banks relative to GDP, *Finance* is the amount of domestic credit offered by the finance sector as a percent of GDP, and *Private* is the amount of domestic credit offered by the private sector scaled by GDP. The control variables include the following: *Ln(GDP/Capita)* is the natural log of GDP per capita. *NetEx* is the difference between exports and imports (in billions of U.S. dollars). *Ln(Save/GDP)* is the natural log of the ratio of gross savings to GDP. *Ln(GovSpend)* is the natural log of the national expenditures (in billions of U.S. dollars). *Ln(CapForm)* is the natural log of capital formation in billions of U.S. dollars. We include year fixed effects and report *t*-statistics in parentheses obtained from robust standard errors.

*, **, and *** denote statistical significance at the 0.10, 0.05, and 0.01 levels, respectively.

Next, we replicate our analysis by estimating Eq (4) using one of three measures of equity market development (*Ln(Equity)*) as the dependent variable. We then estimate Eq (5) using the predicted values on equity market development from the first stage as the independent variable of interest. We report the results of this 2SLS analysis in Table 7. In columns [1] and [2], the potentially endogenous variable is *Ln(Turn)*, which is the natural log of share turnover, or the total dollar volume on all domestic stock exchanges scaled by the market capitalization of all domestic stocks. Consistent with La Porta et al. (1997), the results of the first-stage regression show a positive and significant coefficient on *English Origin*. The predicted value on *Ln(Turn)* in the second-stage regression also produces a positive and significant estimate. In economic terms, a one percent increase in the predicted value of *Ln(Turn)* is associated with a 1.39% increase in pharmaceutical innovation. Similar results are found in columns [3] and [4] and in columns [5] and [6] for the different measures of equity market development. These findings seem to suggest that equity market development influences pharmaceutical innovation, accounting for potential simultaneity bias.

**Table 7:** Pharmaceutical innovation and equity market development–Instrumental variable analysis.

	1^st^ Stage	2^nd^ Stage	1^st^ Stage	2^nd^ Stage	1^st^ Stage	2^nd^ Stage
	[1]	[2]	[3]	[4]	[5]	[6]
*English Origin*	0.2916***		1.0851***		0.8409***	
	(4.36)		(13.09)		(15.15)	
*Ln(Turn)*		1.3860***				
		(3.03)				
*Ln(Vol/GDP)*				0.3193***		
				(3.31)		
*Ln(MCAP/GDP)*						0.4086***
						(3.24)
Adj. R^2^	0.5093	0.2121	0.5865	0.2768	0.4211	0.3047
Controls	Yes	Yes	Yes	Yes	Yes	Yes
N	1,395	1,395	1,625	1,625	1,451	1,451

The table reports the results from estimating the following equations on annual cross-country data using 2SLS:

${Ln(Equity}_{i,t})=\alpha +\gamma_{t}+\beta_{1}{English\;Origin}_{i}+\beta_{2}{Ln(GDP/Capita}_{i,t})+\beta_{3}{NetEx}_{i,t}+\beta_{4}{Ln(Save/GDP}_{i,t})+\beta_{5}{Ln(GovSpend}_{i,t})+\beta_{6}{Ln(CapForm}_{i,t})+\varepsilon_{i,t},$

and

${Ln(PharmaGDP}_{i,t})=\alpha +\gamma_{t}+\beta_{1}{Ln(\hat{Equit}y}_{i,t})+\beta_{2}{Ln(GDP/Capita}_{i,t})+\beta_{3}{NetEx}_{i,t}+\beta_{4}{Ln(Save/GDP}_{i,t})+\beta_{5}{Ln(GovSpend}_{i,t})+\beta_{6}{Ln(CapForm}_{i,t})+\varepsilon_{i,t},$

where the dependent variable in the first stage is the natural log of one of three measures of equity market development. The instrument in the first stage is an indicator variable capturing countries that historically adopted an English Legal Origin (following La Porta et al. 1997)–zero otherwise. The dependent variable in the second stage is the natural log of *Pharma/GDP*, which is the amount of pharmaceutical exports relative to GDP. The independent variable of interest in the second stage is the predicted value on *Ln(Equity)*, which is the natural log of one of three measures of equity market development: *Turn* is equal to total dollar volume (on all domestic stock exchanges) scaled by market capitalization, *Vol/GDP* is the ratio of total dollar volume to GDP, and *MCAP/GDP* is the total market capitalization of all publicly traded stocks in the domestic country as a percent of GDP.The control variables include the following: *Ln(GDP/Capita)* is the natural log of GDP per capita. *NetEx* is the difference between exports and imports (in billions of U.S. dollars). *Ln(Save/GDP)* is the natural log of the ratio of gross savings to GDP. *Ln(GovSpend)* is the natural log of the national expenditures (in billions of U.S. dollars). *Ln(CapForm)* is the natural log of capital formation in billions of U.S. dollars. We include year fixed effects and report *t*-statistics in parentheses obtained from robust standard errors.

*, **, and *** denote statistical significance at the 0.10, 0.05, and 0.01 levels, respectively.

The results in Tables 6 and 7 begin to allow us to draw stronger causal inferences regarding the directional relation between financial market development (credit and equity) and pharmaceutical innovation.

### 3.5. Accounting for potential endogeneity–Adoption of the Euro

In our second attempt to account for the potential endogeneity in our initial set of tests, we examine the pharmaceutical innovation in countries that initially adopted the Euro in 1999, relative to those that did not. Prior research shows that the implementation of the Euro resulted in a positive shock to both the credit market and equity market development (see e.g., [51–53]). We estimate specifications of the following equation on a subsample of European countries, assuming that the adoption of the euro was at least quasi-exogenous to pharmaceutical innovation:

$${Ln(PharmaInnov}_{i,t})=\alpha +\beta_{1}{Euro}_{i}+\beta_{2}{Post}_{t}+\beta_{3}{Euro}_{i}\times {Post}_{t}+\beta_{4}{Ln(GDP/Capita}_{i,t})+\beta_{5}{NetEx}_{i,t}+\beta_{6}{Ln(Save/GDP}_{i,t})+\beta_{7}{Ln(GovSpend}_{i,t})+\beta_{8}{Ln(CapForm}_{i,t})+\varepsilon_{i,t},$$ (6)

where the dependent variable is the natural log of one of three measures of pharmaceutical innovation: *Pharma*, *Pharma/GDP*, *Pharma/Ex*. *Euro* is a categorical variable equal to one if the *i*^th^ country adopted the Euro beginning in 1999 and zero otherwise. *Post* is an indicator variable equal to one for the period from 1999 to 2005 and zero otherwise. The independent variable of interest is the interaction term between *Euro* and *Post*, which is the difference-in-difference estimator. The control variables have been defined previously. We do not include year fixed effects in order to avoid violating the full column rank condition necessary for consistent estimation. The results from estimating Eq (6) are reported in Table 8, with *t*-statistics in parentheses obtained from robust standard errors.

**Table 8:** Pharmaceutical innovation around the adoption of the Euro–difference-in-difference.

	Ln(Pharma)	Ln(Pharma/GDP)	Ln(Pharma/Ex)
	[1]	[2]	[3]
*Euro*	-0.2904	-0.2884	-0.3391*
	(-1.37)	(-1.37)	(-1.88)
*Post*	0.0036	0.0074	-0.0585
	(0.02)	(0.05)	(-0.43)
*Euro×Post*	0.5485**	0.5261**	0.3730*
	(2.26)	(2.18)	(1.73)
*Ln(GDP/Capita)*	0.8432***	0.8267***	0.7248***
	(11.18)	(10.89)	(10.51)
*NetEx*	0.0051**	0.0040*	0.0005
	(2.15)	(1.68)	(0.21)
*Ln(Save/GDP)*	0.0158	-0.0799	-0.4842***
	(0.09)	(-0.46)	(-2.85)
*Ln(GovSpend)*	1.6284***	0.5405***	0.6396***
	(8.52)	(2.96)	(3.66)
*Ln(CapForm)*	-0.7769***	-0.6865***	-0.5825***
	(-3.95)	(-3.65)	(-3.24)
*Constant*	-11.0318***	-10.5091***	-12.2546***
	(-11.24)	(-10.64)	(-13.04)
Adjusted R^2^	0.7974	0.3556	0.3669
Robust SEs	Yes	Yes	Yes
N	442	442	442

The table reports the results from estimating the following equation used an unbalanced panel of European countries during the 12-year period around the adoption of the Euro.

${Ln(PharmaInnov}_{i,t})=\alpha +\beta_{1}{Euro}_{i}+\beta_{2}{Post}_{t}+\beta_{3}{Euro}_{i}\times {Post}_{t}+\beta_{4}{Ln(GDP/Capita}_{i,t})+\beta_{5}{NetEx}_{i,t}+\beta_{6}{Ln(Save/GDP}_{i,t})+\beta_{7}{Ln(GovSpend}_{i,t})+\beta_{8}{Ln(CapForm}_{i,t})+\varepsilon_{i,t},$

where the dependent variable is the natural log of one of three measures of pharmaceutical innovation: *Pharma* is the number of pharmaceutical exports (in $billions), *Pharma/GDP* is the amount of pharmaceutical exports relative to GDP, and *Pharma/Ex* is the amount of pharmaceutical exports relative to total exports. *Euro* is a categorical variable equal to one if the *i*^th^ country adopted the Euro beginning in 1999 and zero otherwise. *Post* is an indicator variable equal to one for the period from 1999 to 2005 and zero otherwise. The independent variable of interest is the interaction term between *Euro* and *Post*, which is the difference-in-difference estimator. The control variables include the following: *Ln(GDP/Capita)* is the natural log of GDP per capita. *NetEx* is the difference between exports and imports (in billions of U.S. dollars). *Ln(Save/GDP)* is the natural log of the ratio of gross savings to GDP. *Ln(GovSpend)* is the natural log of the national expenditures (in billions of U.S. dollars). *Ln(CapForm)* is the natural log of capital formation in billions of U.S. dollars. To avoid the full-rank condition, we do not include year fixed effects. We report *t*-statistics in parentheses obtained from robust standard errors.

*, **, and *** denote statistical significance at the 0.10, 0.05, and 0.01 levels, respectively.

In the first column of Table 8, we report the results from estimating Eq (6) when total pharmaceutical exports is the dependent variable. We find that the total amount of pharmaceutical exports increased by 54.85 percentage points more for European countries that adopted the Euro, relative to those that did not adopt the Euro.

In the second column of Table 8, we report the estimated coefficients from Eq (6) when the ratio of pharmaceutical exports to GDP is the dependent variable. Here, we find that the amount of pharmaceutical exports to GDP increases by 52.61 percentage points more for European countries that adopted the Euro, compared to those that did not adopt the Euro.

In the final column of Table 8, we display the estimated results from Eq (6) when the ratio of pharmaceutical exports to total exports is set as the dependent variable. We find that the amount of pharmaceutical exports to total exports increases by 37.30 percentage points more for European countries that adopted the Euro compared to those that did not adopt the Euro. We note, however, that the interaction term in the third column is only significant at the 0.10 level. To the extent that the adoption of the Euro represents an exogenous shock to financial development in the adopting countries, our analysis suggests that causality flows from credit/equity market development to pharmaceutical innovation and not the other way around.

## 4. Conclusion

This study explores the link between financial development and pharmaceutical innovation. Our analysis is motivated by two streams of literature. The first discusses how pharmaceutical innovation benefits society. A broad literature shows that pharmaceutical innovation improves longevity, lowers healthcare costs, and ultimately increases economic growth. For instance, [4] shows that for every $1 increase in pharmaceutical innovation, hospital-care expenditures decline by $3.65. The second literature shows a surprisingly stark decline in the amount of pharmaceutical innovation over the latter half of the 20^th^ century. Given the benefits associated with innovation, and the unfortunate decline in the rate of innovation over time, discovering factors that influence pharmaceutical innovation becomes important.

Using a broad cross-country sample from 1989 to 2016, this study shows that, in general, financial development increases pharmaceutical innovation. In particular, credit market development leads to higher pharmaceutical exports across countries, and to a lesser extent, equity market development also contributes to greater levels of pharmaceutical innovation. To draw stronger causal inferences, we estimate a series of 2SLS regressions using the legal origin as an instrumental variable and find that, controlling for potential endogeneity, financial market development influences pharmaceutical innovation. We also use the adoption of the Euro as a natural experiment and conduct a series of difference-in-difference tests around the implementation of the Euro. To the extent that the implementation of the Euro created an exogenous shock to the level of financial development, we expect to observe higher rates of pharmaceutical innovation in countries that adopted the Euro vis-à-vis countries that did not adopt the Euro during the post-implementation period. The empirical results confirm this expectation. These tests allow us to begin to speak to the direction of causation, which seems to flow from financial development to pharmaceutical innovation, and not vice-versa.
